# Increased malignancy of oral squamous cell carcinomas (oscc) is associated with macrophage polarization in regional lymph nodes – an immunohistochemical study

**DOI:** 10.1186/1471-2407-14-522

**Published:** 2014-07-21

**Authors:** Falk Wehrhan, Maike Büttner-Herold, Peter Hyckel, Patrick Moebius, Raimund Preidl, Luitpold Distel, Jutta Ries, Kerstin Amann, Christian Schmitt, Friedrich W Neukam, Manuel Weber

**Affiliations:** 1 Department of Oral and Maxillofacial Surgery, Friedrich-Alexander University Erlangen-Nürnberg, Glueckstrasse 11, 91054 Erlangen, Germany; 2 Institute of Pathology, Department of Nephropathology, Friedrich-Alexander University Erlangen-Nürnberg, Krankenhausstraße 12, 91054 Erlangen, Germany; 3 Department of Oral and Maxillofacial Surgery, University of Jena, Erlanger Allee 101, 07749 Jena, Germany; 4 Department of Radiation Oncology, Friedrich-Alexander University Erlangen-Nürnberg, Universitätsstraße 27, 91054 Erlangen, Germany

**Keywords:** Oral squamous cell carcinoma, Oral cancer, Lymph node, Macrophage polarization, Peripheral tolerance, oscc, M1, M2

## Abstract

**Background:**

It is largely accepted that specific immunological parameters in solid malignancies are associated with patient’s prognosis. Recently a correlation of macrophage polarization with histomorphological parameters could also be shown in oral squamous cell carcinoma (oscc). The observed tumor derived peripheral immune tolerance could be associated with the macrophage polarization in regional tumor draining lymph nodes.

So far there are no studies analyzing the macrophage polarization in cervical lymph nodes of oscc patients. In the present study we aimed to correlate macrophage polarization in different anatomical lymph node compartments of patients diagnosed with oscc with histopathologic parameters of the primary tumor (T-, N-, L-, V-, Pn-status, grading).

**Methods:**

Tumor free (n = 37) and metastatic (n = 17) lymph nodes of T1 and T2 oscc patients were processed for immunohistochemistry to detect CD68, CD11c, CD163 and MRC1 positive cells. Samples were digitized using whole slide imaging and the number of cells expressing the aforementioned markers in the region of interest quantitatively analyzed.

**Results:**

The malignancy of the primary tumor (defined by T-, L-, Pn-status, grading) correlated with the lymph node macrophage polarization. L1 and Pn1 tumor cases displayed a significantly (p < 0.05) decreased M1 and increased M2 polarization in the sinus of the lymph nodes. G3 cases presented a significantly (p < 0.05) increased M2 polarization in the sinus compared to G2 cases. T2 tumors had significantly (p < 0.05) increased M2 polarization in the interfollicular zone of regional lymph nodes compared to T1 tumors. Metastatic and non-metastatic lymph nodes did not differ regarding their macrophage polarization.

**Conclusions:**

The current study revealed for the first time an influence of oscc on the macrophage polarization in regional lymph nodes. Markers of malignant behavior in the primary tumor were associated with a shift of macrophage polarization in lymph nodes from the anti-tumoral M1 type to the tumor-promoting M2 type. As tumor free and metastatic lymph nodes did not differ in terms of their macrophage polarization pattern, there must be other factors influencing the location for lymph node metastasis formation.

## Background

Despite the decline in prevalence of smoking in industrial countries, there is no reduction in oral squamous cell carcinoma (oscc) incidence noticeable [1]. It is currently the 8^th^ most frequent tumor worldwide [1-3]. Although, nowadays there are advances in surgical treatment options, the overall prognosis of this specific cancer type could not be improved during the last 30 years [4]. The occurrence of local lymphogenic metastasis is one of the strongest prognostic determinants in oscc [5,6]. In many cases, oral cancer is diagnosed at a metastatic stage causing therapeutic difficulties [1,7].

The influence of immunological parameters on the prognosis of oscc has already been discussed in the 1970^th^ and 1980^th^[8,9]. The non-specific immune parameter phytohemagglutinin (PHA) –reactivity, measured in patient’s serum, showed a significant correlation with the occurrence of lymph node metastasis [8]. The lymphokine release measured after mitogenic PHA stimulation is an indicator for the degree of cellular immune reactivity [8]. Low PHA-reactivity is associated with the tolerance inducing M2 macrophage polarization [10]. In the further course, immunological contributes to oscc pathogenesis have been neglected.

Nowadays, literature is conclusive about the fact that tumor immunology plays an important role for local lymph node metastasis [11-13]. In our current understanding of the formation of lymphogenic metastases, the concept of peripheral tolerance plays an important role. The capacity of malignancies to evade host immune defense reactions and to establish a state of peripheral immune tolerance was initially described in highly immunogenic tumors like melanomas [14,15]. It was shown that early stage melanomas have the peculiarity to communicate with tumor draining lymph nodes and thus prepare them for the acceptance and growth of metastases [14]. In metastatic lymph nodes there is an immediate proximity between tumor cells and various types of leukocytes. However, instead of an anti-tumoral immunity, a state of peripheral immune tolerance can be observed [14,16]. Besides malignant melanoma, the relevance of immunological markers in tumor tissue could be shown in solid malignancies [11,17-19]. Several studies also demonstrated the importance of the immune system for oscc progression [10,17,20-25].

Results of our group reveal a correlation of macrophage polarization in oscc specimens with histomorphological parameters. An increased M2 polarization in the epithelial tumor compartment was associated with the occurrence of lymph node metastasis [26].

The human papilloma virus (HPV) associated oscc underlines the importance of the immune system for the pathogenesis of oral cancer. It is assumed that the favorable prognosis in HPV positive cases might be associated with an immune response against viral antigens [25,27,28]. This observation outlines the potential ability of the immune system to engage oral cancer.

The lymph flow from tumors is increased compared to normal tissue [16]. Immune tolerance in regional lymph nodes is a prerequisite for the formation of lymph node metastasis [14]. Macrophages and dendritic cells migrate from peripheral tissue to the lymph nodes and orchestrate the balance between tolerance and immunity [16]. M1 macrophages are responsible for elimination of pathogens, tissue destruction and tumor resistance [29-31]. In contrast, M2 cells have immunoregulatory properties and are associated with tissue remodeling, angiogenesis and tumor progression [11,29-34].

CD68 is the best established generic macrophage marker [11,24,35,36]. M1 macrophages are commonly identified by staining the CD11c antigen [32,35,37,38], M2 macrophages express the antigens CD163 [33,36,39,40] and MRC1 [32,39,41].

Additionally, the relative proportion of the M1 and M2 markers should also be considered. In analogy to other recent studies dealing with macrophage polarization in human cancer tissues [19,26,42,43], we aimed to analyze the ratios between the stained markers.

So far no studies exist quantifying the macrophage polarization in regional tumor draining lymph nodes of solid malignancies. Therefore, the aim of this study is to clarify if the macrophage polarization in regional lymph nodes and lymph node metastases of small (pT1, pT2) primary oscc is correlated with histopathologic parameters like TNM-status, grading, lymph-vessel-, and perineural-infiltration. To this extend immunohistochemical analysis of the specimens was performed in different lymph node compartments (interfollicular zone, lymph node sinus and perisinusoidal zone) using a computer assisted quantitative cell counter.

## Methods

### Patients and tissue harvesting

Resected lymph nodes of 37 patients, histologically diagnosed with primary oscc, were analyzed in this study. 18 patients had proven lymph node metastasis (pN+), 19 were free of metastases (pN0). Metastasis free lymph nodes were evaluated in each subject no matter of the lymph node status. Additionally, a positive lymph node in each pN + case was analyzed. Thus, a total of 54 lymph nodes were examined (17 with and 37 without metastasis). All included patients were treated in 2011 at the Department of Oral and Maxillofacial Surgery of the University Hospital Erlangen. The study protocol was approved by the ethical committee of the University of Erlangen-Nuremberg (Ref.-No. 45_12 Bc). The specimens used in this retrospective study were obtained from tissue samples of consecutive patients collected for routine histopathologic diagnosis. The specimens were archival samples from the Comprehensive Cancer Center Erlangen-EMN. Each included lymph node was judged as a representative lymph node. Besides the main diagnosis of oscc following additional inclusion criteria were defined: pT1 and pT2 tumors, no restrictions in the grading of the tumor, no adjuvant preoperative radio- or chemotherapy and no organ metastasis at the time of diagnosis. Tumor status (T-, N-, L-, Pn-status) and tumor grading was determined by the routine pathological analysis of the resected tumor and lymph node specimens.

Only oscc lymph nodes of patients with pT1 and pT2 were considered, because the tumors of these patients are characterized by a better resectability and prognosis compared to larger pT3 and pT4 tumors [44]. Furthermore, T4 tumors might have a special immunological microenvironment due to their direct contact to bone marrow stem cells.

Patients with former radio- or chemotherapy as well as pT3 and pT4 tumors were excluded. There were no study related changes in patient’s treatments.

In terms of the primary tumor location patient collective (n = 37) consisted of 9 patients with a tumor of the tongue, 15 patients with a tumor of the floor of the mouth, 8 of the alveolar crest, 3 of the palate and 2 of the cheek.

The average age of the patients (24 males and 13 females) was 62 years. The pathohistological classified T-, N-, L-, Pn-status was T1 in 17 cases and T2 in 20 cases, N0 in 19 cases and N + in 18 cases, L0 in 26 cases and L1 in 10 cases and Pn0 in 23 cases and Pn1 in 10 cases. Some cases could not be clearly classified regarding their L- and Pn-status. One case was graded as G1, 28 as G2 and 8 as G3.

### Immunohistochemical stainings

The formalin-fixed, paraffin-embedded tissue samples were sliced in consecutive sections of 2 μm thickness with a rotation microtome (Leica, Nussloch, Germany), then dewaxed in xylole and rehydrated in graded propanol prior to immunohistochemical staining. Immunohistochemical staining was performed with the LSAB (labeled streptavidin-biotin) method and an automated staining device (Autostainer plus, Dako Cytomation, Hamburg, Germany). The staining kit (Dako Real, Cat. K5001, Dako Cytomation) was used according to manufacturer’s instructions. Proteins were detected by incubating tissues in the autostainer (21°C, 30 min) with specific antibodies.

The following primary antibodies were used: anti-CD11c (ab52632, clone EP1347y, Abcam, Cambridge, UK), anti-CD68 (11081401, clone KP1, Dako, Hamburg, Germany), anti-CD163 (MAB1652, clone K20-T, Abnova, Taipei City, Taiwan) and anti-MRC1 (H00004360-1102, clone 5C11, Abnova).

As secondary antibody the biotinylated immunoglobulins were used for all samples. Stained portions were visualized with the DAB + solution (Dako Cytomation), localized by biotin-associated activation of the secondary antibodies. This was followed by incubation in hematoxylin (Dako Cytomation) for counterstaining of the nucleus. Two consecutive tissue samples were processed per immunohistochemical staining; one served as a negative control in each case (identical treatment, but replacement of the primary antibody with an IgG-isotype of the primary antibody). A positive control sample that was known to stain positive for a given antibody was included in each series.

### Quantitative immunohistochemical analysis

**Figure 1:**
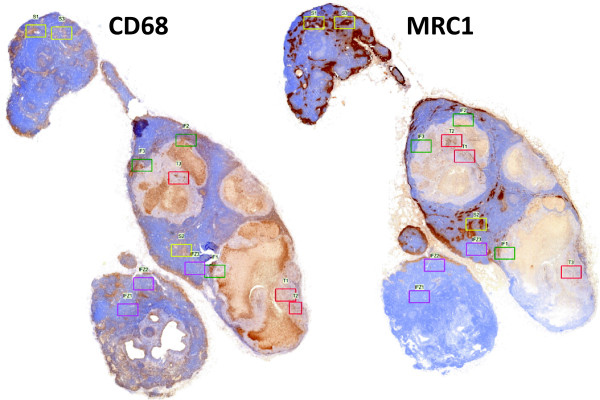
**Selection of corresponding fields of view in a lymph node metastasis.** The figure shows exemplarily two sequential slides of a lymph node metastasis specimen virtually microscoped. On the left side CD68 positive cells and on the right side MRC1 positive cells are stained. Visual fields for cell count are shown: lymph node sinus (LN sinus) - *yellow*, lymph node interfollicular zone (LN IFZ) - *blue*, metastatic tumor (TU) - *red*, tumor invasion front (TU Front) - *green*. Corresponding fields of view have been selected for LN sinus, LN IFZ and TU Front. In TU fields of view for each marker the region of the highest expression was selected.

The lymph node sections were completely scanned and digitized using the method of “whole slide imaging”. The scanning procedure was performed in cooperation with the Institute of Pathology of the University of Erlangen using a Zeiss MIRAX MIDI Scanner (Zeiss, Jena, Germany). All samples were analyzed on a computer (Panoramic MIRAX viewer, Zeiss, Jena, Germany). Quality controls were performed under a bright-field microscope (Zeiss Axioskop and Axiocam 5, at 100–400 × magnification). HE-stained sections of all samples were examined by a pathologist to ensure that all samples contained representative lymph nodes.For each sample, the following two different categories of visual fields were selected: The lymph node sinus and the interfollicular zone (Figure 1). For each category, three visual fields per section showing the highest infiltration rate of CD68 expressing cells were selected. For the other markers (CD11c, CD163 and MRC1) the corresponding fields of view were selected (Figure 1) using multi-monitor virtual microscopy (Panoramic MIRAX viewer, Zeiss). Consequently, 24 fields of view were assessed for cell counting for each specimen.

The complete area of all three visual fields of one category was between 1.1 mm^2^ and 1.5 mm^2^.

The images showing the visual fields were imported into Biomas (MSAB, Erlangen, Germany) for cell counting. In the visual fields of the sinuses two regions of interest were defined in Biomas software: The perisinusoidal zone and the lymph node sinus (LN sinus).

A quantitative analysis was performed to determine the infiltration levels of CD11c-, CD68-, CD163- and MRC1-positive cells in the lymph node sinus (LN sinus), the perisinusoidal zone and the interfollicular zone (LN IFZ). All positive cells in the aforementioned compartments were manually counted. Cell density per mm^2^ was automatically calculated by the Biomas software. Only cells with a monocytoid/macrophage-like morphology were counted. Cell counting was performed in a blinded manner by research fellows familiar with tissue morphology analyses and immunohistochemical methods.

### Statistical analysis

In order to analyze immunohistochemical staining and spatial expression patterns, the cell count per mm^2^ was determined as the number of positive cells per mm^2^ of the specimen. Multiple measurements were pooled for each sample group prior to analysis. The results are expressed as the median, the interquartile range (IQR), standard deviation (SD) and range. Box plot diagrams represent the median, the interquartile range, minimum (Min) and maximum (Max).

Two-sided, adjusted p-values ≤ 0.05 were considered to be significant. Analyses were performed with SPSS 21 for Mac OS (IBM Inc, New York, USA).

## Results

### General morphological considerations

**Figure 2:**
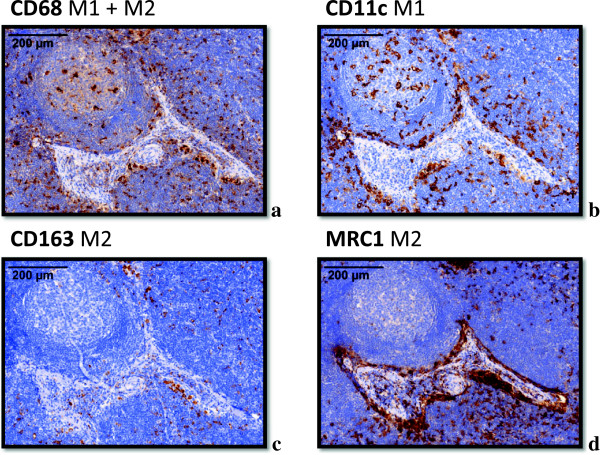
**Typical expression pattern of the macrophage markers CD68, CD11c, CD163 and MRC1 in a lymph node.** Exemplary fields of view (original magnification 40x) showing the typical expression pattern of the stained macrophage markers CD68, CD11c, CD163 and MRC1 in lymph node specimens. **a) **CD68: The marker shows a cytoplasmatic staining. The shape of the stained cells includes predominantly round cells, but some cells also show a spindle-shape. CD68 is expressed in the follicles, in the interfollicular zone and in the sinus. Besides a distinct staining of the macrophages, in lymph node metastasis specimens a pale staining of tumor cells was also detectable. **b)** CD11c: The marker shows a cytoplasmatic expression pattern with an accentuation of the plasma membrane. The shape of the stained cells includes predominantly round cells. CD11c expressing cells were mainly found in the follicles and in the interfollicular zone. **c)** CD163: The marker shows a cytoplasmatic expression pattern with an accentuation of the plasma membrane. The shape of the stained cells includes predominantly spindle-shaped cells. CD163 expression can predominantly be found in the lymph node sinus. The follicles are largely missing CD163 expressing cells. **d)** MRC1: The marker shows a cytoplasmatic expression pattern with an accentuation of the plasma membrane. The shape of the stained cells includes predominantly spindle-shaped cells. MRC1 expression can predominantly be found in the lymph node sinus. The follicles are largely missing MRC1 expressing cells.

All specimens were analyzed in a virtual microscope system. Except for CD11c, all stained markers displayed a significantly (p < 0.001) higher number of positive cells in the lymph node sinus compared to the other analyzed compartments. In the sinus, the distribution of macrophages seemed to be quite homogeneous. In the interfollicular zone some spots of increased infiltration were visible.CD68 staining was used to define the visual field subsequently analyzed for all stainings (Figure 1). CD68 positive cells could be identified in all lymph node compartments including the follicles. A cytoplasmatic expression was found. The shape of the stained cells included predominantly round cells, but some cells also showed a spindle-shape (Figure 2). CD11c expressing M1 macrophages were found in all lymph node compartments. The distribution pattern of the CD11c expressing cells was more comparable to CD68 positive cells than to the distribution of M2 marker expressing cells. Compared to the other analyzed compartments there was no significant accumulation of CD11c expressing cells in the lymph node sinus. The CD11c expressing cells were stained cytoplasmatically as well as membrane-bound and had a round shape (Figure 2). The M2 markers CD163 and MRC1 showed an accentuation of expression in the lymph node sinus. In contrast to CD68 and CD11c, in the follicles no expression of the M2 markers was detectable. CD163 and MRC1 staining was visible in the cytoplasm and membrane-bound. Most of the stained cells had a spindle-shape (Figure 2).

### Macrophage polarization and lymph node metastasis

Comparing the macrophage polarization in the tumor free lymph nodes and in the metastatic nodes, no statistical significant differences could be detected. There was also no tendency of any parameter to be different between the groups. This finding supports the hypothesis that metastatic and non-metastatic lymph nodes do not differ regarding their macrophage polarization.

### Macrophage polarization and the N-status

Macrophage polarizations in tumor free lymph nodes of patients with nodal metastases (pN+) were compared to patients without lymph node metastases (pN0). No correlation of the macrophage polarization in tumor free lymph nodes with the pN-status was observed.

### Macrophage polarization and the L-status

**Figure 3:**
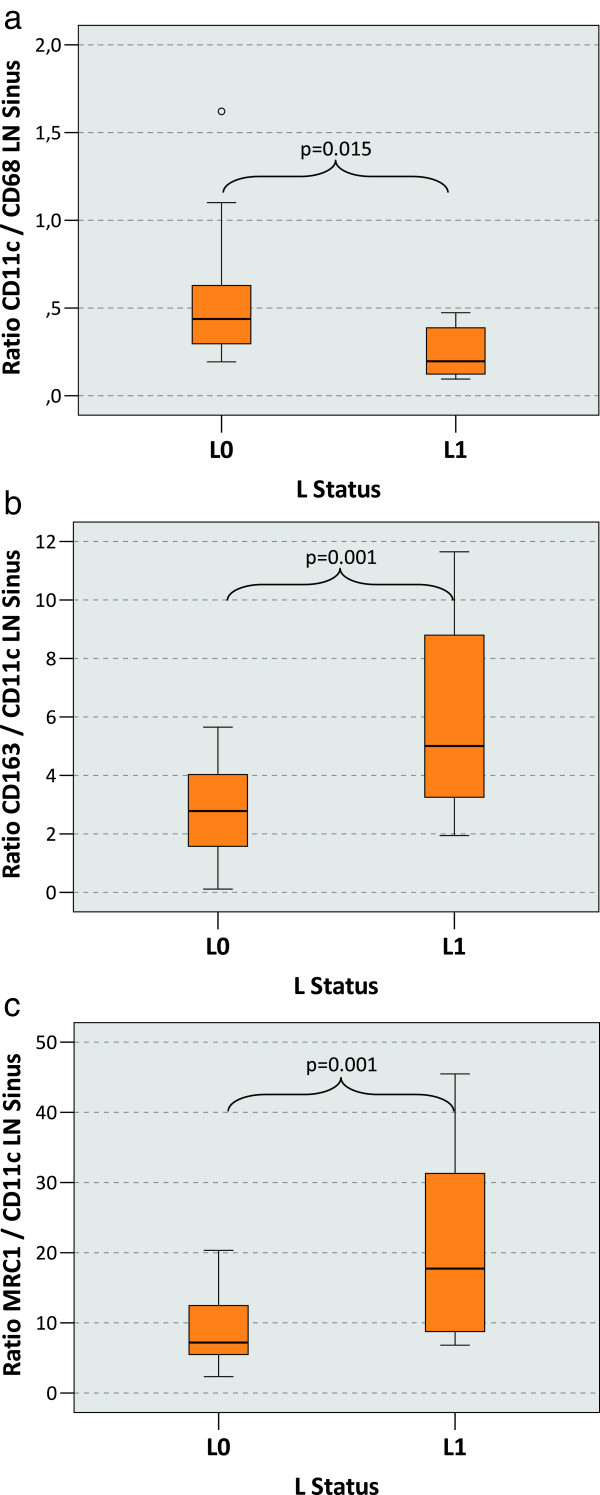
**Macrophage polarization in the lymph node sinus depending on the L-status of the primary tumor. a)** The figure shows the ratio between the CD11c cell count and the CD68 cell count in the lymph node sinus (LN sinus) as indicator of M1 polarization. Tumor draining lymph nodes free of metastasis have been examined. P-values generated by the ANOVA-test are indicated. A significantly decreased CD11c/CD68 ratio in the lymph node sinus can be found in cases with lymph vessel infiltration at the primary tumor site (L1) compared to L0 cases. **b)** The figure shows the ratio between the CD163 cell count and the CD11c cell count in the lymph node sinus (LN sinus) as indicator of M2 polarization. Tumor draining lymph nodes free of metastasis have been examined. P-values generated by the ANOVA-test are indicated. A significantly increased CD163/CD11c ratio in the lymph node sinus can be found in in cases with lymph vessel infiltration at the primary tumor site (L1) compared to L0 cases. **c)** The figure shows the ratio between the MRC1 cell count and the CD11c cell count in the lymph node sinus (LN sinus) as indicator of M2 polarization. Tumor draining lymph nodes free of metastasis have been examined. P-values generated by the ANOVA-test are indicated. A significantly increased MRC1/CD11c ratio in the lymph node sinus can be found in in cases with lymph vessel infiltration at the primary tumor site (L1) compared to L0 cases.

A correlation of the macrophage polarization in tumor free regional lymph nodes with histologically defined lymph vessel infiltration status (L-status) of the primary tumor could be identified. The ratio between the CD11c positive cells (predominantly M1 macrophages) and CD68 positive cells (all macrophages) represents the degree of M1 polarization. In the lymph node sinus (LN sinus) the CD11c/CD68 ratio was significantly (p = 0.015) lower in the L1 cases (median value 0.20) compared to L0 cases (median value 0.44) (Table 1, Figure 3a).

**Table 1:** Ratio of macrophage marker expression depending on the T-status, N-status, L-status, Pn-status and grading (cell count in the sinus and interfollicular zone (IFZ) of tumor free lymph nodes)

**Ratio**		**CD11c/CD68 LN sinus**		**CD163/CD68 LN sinus**		**MRC1/CD68 LN sinus**		**CD163/CD11c LN sinus**		**MRC1/CD11c LN sinus**		**MRC1/CD68 LN IFZ**		**MRC1/CD11c LN IFZ**	
		**Median**	**SD**	**Median**	**SD**	**Median**	**SD**	**Median**	**SD**	**Median**	**SD**	**Median**	**SD**	**Median**	**SD**
***T-status***	*n*														
*T1*	*17*	0.33	0.23	1.09	0.63	4.03	1.72	3.81	2.39	10.07	11.57	0.33	0.19	0.36	0.21
*T2*	*19*	0.39	0.36	0.99	0.38	3.36	1.55	3.25	2.81	8.76	8.56	0.6	0.29	0.51	0.4
*p-value*		0.455		0.464		0.384		0.608		0.328		0.027		0.041	
***N-status***	*n*														
*N0*	*19*	0.33	0.25	1.08	0.58	3.39	1.92	3.66	2.23	12.46	8.60	0.32	0.24	0.36	0.39
*N+*	*17*	0.36	0.36	1.02	0.43	3.65	1.25	3.11	3.00	7.39	11.78	0.60	0.27	0.49	0.29
*p-value*		0.902		0.963		0.458		0.725		0.958		0.073		0.922	
***L-status***	*n*														
*L0*	*26*	0.44	0.32	0.99	0.60	3.36	1.67	2.78	1.6	7.19	5.24	0.45	0.25	0.49	0.36
*L1*	*10*	0.2	0.14	1.09	0.17	3.52	0.93	5.01	3.35	17.73	13.36	0.35	0.30	0.33	0.28
*p-value*		0.015		0.747		0.813		0.001		0.001		0.46		0.24	
***Pn-status***	*n*														
*Pn0*	*23*	0.44	0.33	0.98	0.49	3.22	1.72	2.78	1.58	9.24	5.35	0.44	0.24	0.50	0.37
*Pn1*	*10*	0.23	0.14	1.11	0.13	3.66	0.80	5.01	3.35	17.73	13.32	0.37	0.33	0.40	0.28
*p-value*		0.032		0.717		0.951		0.001		0.002		0.981		0.278	
***Grading***	*n*														
*G1*	*1*	-	-	-	-	-	-	-	-	-	-	-	-	-	-
*G2*	*28*	0.36	0.31	1.02	0.55	3.39	1.59	2.99	1.96	9.24	8.80	0.42	0.27	0.38	0.36
*G3*	*8*	0.26	0.25	1.11	0.36	3.84	1.91	5.39	3.65	17.73	12.39	0.37	0.27	0.46	0.33
*p-value*		0.225		0.779		0.696		0.048		0.090		0.674		0.787	

Table 1 shows the ratio of macrophage marker expression in the sinus and the interfollicular zone (IFZ) of tumor free lymph nodes in cases with different pathohistologic classifications (T1 and T2, N0 and N+, L0 and L1, Pn0 and Pn1, G1 – G3). Values represent the median, standard deviation (SD) and p-value (ANOVA).

The degree of M2 polarization can be described by the ratio of M2 markers CD163 or MRC1 and CD11c. In the LN sinus the CD163/CD11c ratio was significantly (p = 0.001) higher in the L1 cases (median value of 5.01) than in the L0 cases (median value of 2.78) (Table 1, Figure 3b). A similar proportion was revealed by the MRC1/CD11c ratio. With a median value of 17.73 there was a significantly (p = 0.001) higher ratio in L1 cases compared to L0 cases with a median value of 7.19 (Table 1, Figure 3c).

Ratios of other parameters did not correlate with the L-status. In summary, the relative ratio of M1 vs. M2 polarized macrophages showed a significant shift towards M2 polarization in the LN sinus of L1 cases.

### Macrophage polarization and the Pn-status

**Figure 4:**
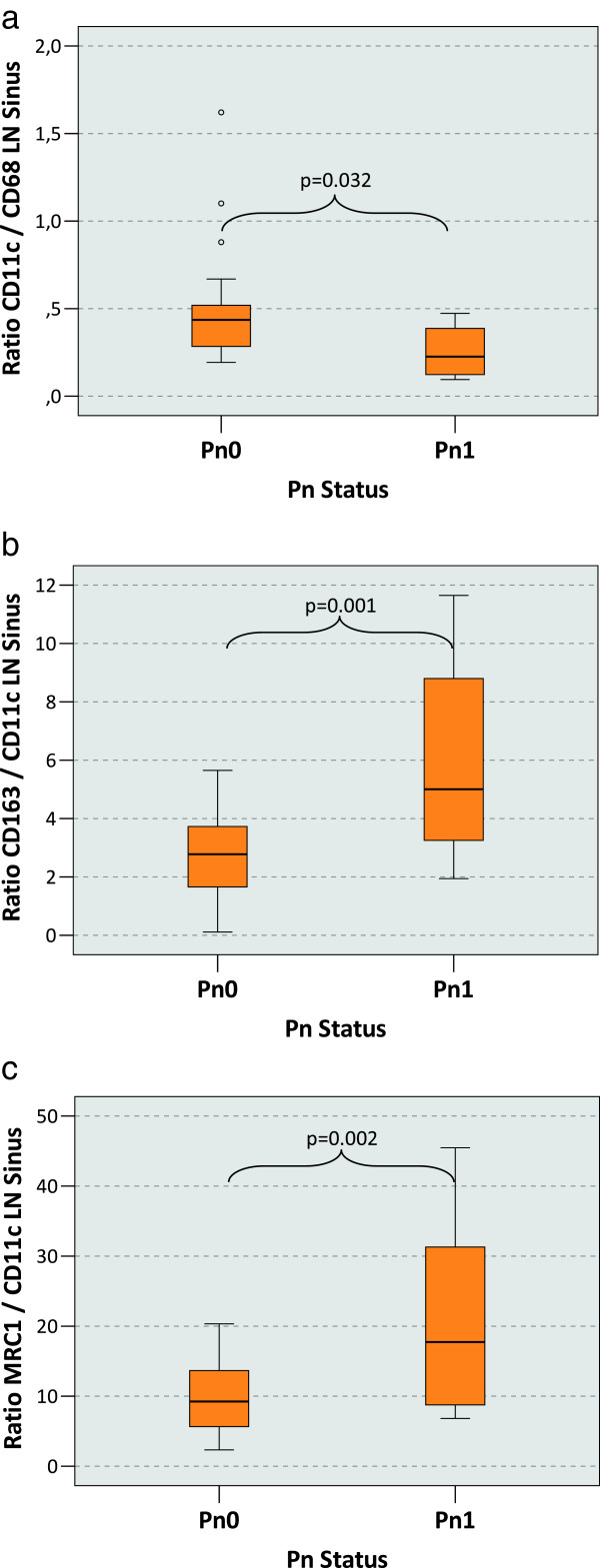
**Macrophage polarization in the lymph node sinus depending on the Pn-status of the primary tumor. a)** The figure shows the ratio between the CD11c cell count and the CD68 cell count in the lymph node sinus (LN sinus) as indicator of M1 polarization. Tumor draining lymph nodes free of metastasis have been examined. P-values generated by the ANOVA-test are indicated. A significantly decreased CD11c/CD68 ratio in the lymph node sinus can be found in cases with perineural infiltration at the primary tumor site (Pn1) compared to Pn0 cases. **b)** The figure shows the ratio between the CD163 cell count and the CD11c cell count in the lymph node sinus (LN sinus) as indicator of M2 polarization. Tumor draining lymph nodes free of metastasis have been examined. P-values generated by the ANOVA-test are indicated. A significantly increased CD163/CD11c ratio in the lymph node sinus can be found in cases with perineural infiltration at the primary tumor site (Pn1) compared to Pn0 cases. **c)** The figure shows the ratio between the MRC1 cell count and the CD11c cell count in the lymph node sinus (LN sinus) as indicator of M2 polarization. Tumor draining lymph nodes free of metastasis have been examined. P-values generated by the ANOVA-test are indicated. A significantly increased MRC1/CD11c ratio in the lymph node sinus can be found in in cases with perineural infiltration at the primary tumor site (Pn1) compared to Pn0 cases.

Correlation of perineural infiltration status (Pn-status) of the primary tumor with lymph node macrophage polarization was similar to the L-status. In the LN sinus the CD11c/CD68 ratio was significantly (p = 0.032) lower in the Pn1 cases (median value 0.23) than in the Pn0 cases (median value 0.44) (Table 1, Figure 4a). The CD163/CD11c resp. the MRC1/CD11c ratio was significantly higher in the Pn1 cases with 5.01 resp. 17.73 (p = 0.001 resp. 0.002) than in the Pn0 cases (2.78 resp. 9.24) (Table 1, Figure 4b and c).

Ratios of other parameters did not correlate with the Pn-status. In summary, the relative ratio of M1 vs. M2 polarized macrophages showed a significant shift towards M2 polarization in the LN sinus of Pn1 cases.

### Macrophage polarization and the grading

**Figure 5:**
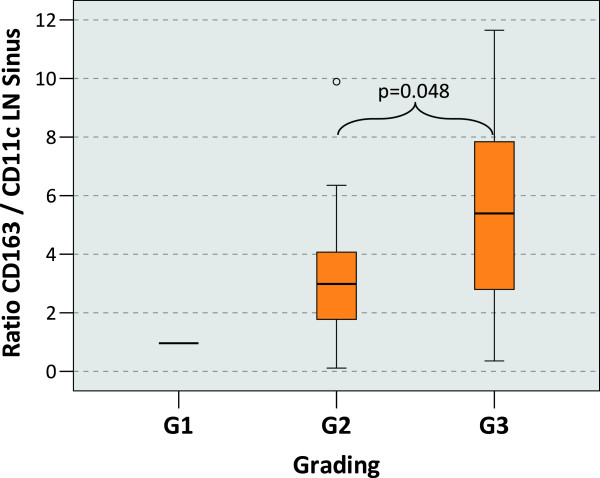
**Macrophage polarization in the lymph node sinus depending on the grading of the primary tumor.** The figure shows the ratio between the CD163 cell count and the CD11c cell count in the lymph node sinus (LN sinus) as indicator of M2 polarization. Tumor draining lymph nodes free of metastasis have been examined. P-values generated by the ANOVA-test are indicated. A significantly increased CD163/CD11c ratio in the lymph node sinus can be found in in cases with Grading 3 (G3) of the primary tumor compared to cases with Grading 2 (G2).

Macrophage polarization in tumor free regional lymph nodes showed a correlation with the histopathological grading of the primary tumor. High grade cases (G3) showed a significantly (P = 0.048) increased relative M2 polarization in the LN sinus compared to intermediate grade (G2) cases. This was shown by the median CD163/CD11c ratio of 2.99 in the G2 and of 5.39 in the G3 cases (Table 1, Figure 5). No other parameters correlated with the tumor grading.

### Macrophage polarization and the T-status

**Figure 6:**
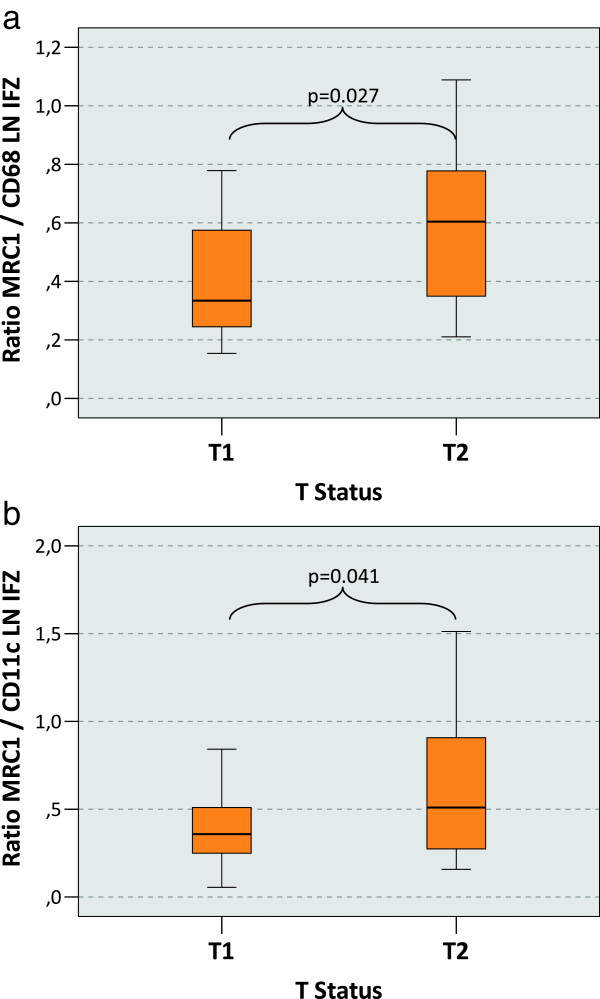
**Macrophage polarization in the lymph node interfollicular zone depending on the T-status of the primary tumor. a)** The figure shows the ratio between the MRC1 cell count and the CD68 cell count in the interfollicular zone of the lymph node (LN IFZ) as indicator of M2 polarization. Tumor draining lymph nodes free of metastasis have been examined. P-values generated by the ANOVA-test are indicated. A significantly increased MRC1/CD68 ratio in the lymph node sinus can be found in pT2 cases compared to pT1 cases. **b)** The figure shows the ratio between the MRC1 cell count and the CD11c cell count in the interfollicular zone of the lymph node (LN IFZ) as indicator of M2 polarization. Tumor draining lymph nodes free of metastasis have been examined. P-values generated by the ANOVA-test are indicated. A significantly increased MRC1/CD11c ratio in the lymph node sinus can be found in pT2 cases compared to pT1 cases.

A correlation between the macrophage polarization in the interfollicular zone (IFZ) of local tumor free lymph nodes and the T-status of the primary tumor could be shown. In contrast to L-status, Pn-status and grading, there was no correlation of the T-status with macrophage polarization in the sinuses. The MRC1/CD68 ratio in the IFZ was significantly (p = 0.027) higher in T2 cases (median value 0.60) compared to the T1 cases (median value 0.33) (Table 1, Figure 6a). An analogous effect was notable considering the MRC1/CD11c ratio in the IFZ. This ratio was also significantly (p = 0.041) higher comparing the T2 cases (median value 0.51) with the T1 cases (median value 0.36) (Table 1, Figure 6b). Ratios of the other parameters did not correlate with the T-status.

### Macrophage polarization and the L-status in tumor free and metastatic lymph nodes

We did not see any differences regarding the macrophage marker expression in tumor free and metastatic lymph nodes. In our further analysis all regional lymph nodes (metastatic and non metastatic) are considered as one group. In analogy to the findings in tumor free lymph nodes a significantly decreased CD11c/CD68 and a significantly increased CD163/CD11c and MRC1/CD11c ratio was detected in the L1 cases compared to the L0 cases. Additionally significant differences in the absolute expression of all analyzed macrophage markers were apparent in the LN sinus.

**Figure 7:**
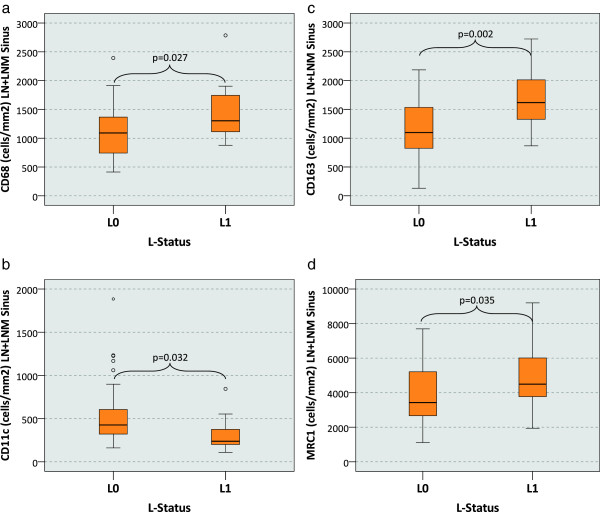
**Macrophage cell count (cells/mm**^**2**^**) in tumor free and metastatic lymph nodes depending on the L-status of the primary tumor.** The figure shows the median macrophage cell count (cells/mm^2^ specimen area) in the lymph node sinus (LN sinus) depending on the lymph vessel infiltration status of the primary tumor (L0 vs. L1). Both groups of tumor draining lymph nodes have been examined (lymph nodes free of metastasis and metastatic lymph nodes). P-values generated by the ANOVA-test are indicated. **a)** CD68 cell count: A significantly increased count can be observed in the L1 cases. **b)** CD11c cell count: A significantly decreased count can be observed in the L1 cases. **c)** CD163 cell count: A significantly increased count can be observed in the L1 cases. **d)** MRC1 cell count: A significantly increased count can be observed in the L1 cases.

In LN sinuses the CD68 cell count was significantly (p = 0.027) higher (median value of 1302 cells/mm^2^) in the L1 cases than in the L0 cases (median value of 1091 cells/mm^2^) (Table 2, Figure 7a). An inverse relationship was apparent analyzing the CD11c expression. With a median value of 238 cells/mm^2^ in L1 cases compared to 423 cells/mm^2^ in L0 cases the sinusoidal CD11c expression was significantly (p = 0.032) lower (Table 2, Figure 7b).

**Table 2:** **Macrophage marker expression (cells/mm**^**2**^**) in L0 and L1 cases (cell count in the sinus of tumor free lymph nodes and metastatic lymph nodes)**

**Marker**		**CD68 LN sinus**		**CD11c LN sinus**		**CD163 LN sinus**		**MRC1 LN sinus**	
		**Median IQR**	**SD**	**Median IQR**	**SD**	**Median IQR**	**SD**	**Median IQR**	**SD**
*L-status*	*n*								
*L0*	*37*	1091	439	423	366	1098	525	3608	1755
		658		299		739		2700	
*L1*	*15*	1302	501	238	189	1618	539	4493	2058
		686		198		793		2495	
*p-value*		0.027		0.032		0.002		0.035	

Table five shows the expression of macrophage markers in the lymph node sinus in L0 and L1 cases. Values represent the median, interquartile range (IQR), standard deviation (SD) and p-value (ANOVA).

In contrast, M2 marker expression in the LN sinus was significantly increased in the L1 cases. CD163 resp. MRC1 expression was significantly (p = 0.002 resp. 0.035) higher in L1 cases (1618 resp. 4493 cells/mm^2^) than in L0 cases (1098 resp. 3608 cells/mm^2^) (Table 2, Figure 7c and d).

In summary, L1 cases show a significantly decreased M1 and a significantly increased M2 polarization in the LN sinus. These correlations are apparent in the metastatic as well as in the tumor free regional lymph nodes.

## Discussion

In the present study an association of macrophage polarization in the regional lymph nodes of oral squamous cell carcinomas with parameters of malignancy and invasiveness (T-, L-, Pn-status, grading) of the primary tumor was noted. Markers of malignant behavior in the primary tumor were associated with a shift of macrophage polarization in lymph nodes from the anti-tumoral M1 type to the tumor-promoting M2 type.

Oscc cases with lymph vessel infiltration at the primary tumor site (L1) showed an increased M2 and a decreased M1 polarization in the sinuses of the regional lymph nodes. This finding might indicate that malignant cell infiltration into the lymph vessels leads to a shift in sinusoidal macrophage polarization towards the tumor-promoting M2 type.

Current literature indicates that carcinomas access the lymphatic system by triggering lymph vessel proliferation and invade into the lymph vessels. Thereby carcinoma cells establish a physical connection to the local tumor draining lymph nodes [16].

Since the lymph node sinus represents a direct connection to the afferent lymph vessels, this is the anatomical compartment of the lymph node with first contact to afferent lymph. Tumor derived factors might influence the macrophage physiology in the sinus polarizing them into a M2 state [16]. Sinusoidal M2 macrophages might contribute to a tumor derived peripheral immune tolerance and could facilitate metastatic growth.

In cases with perineural infiltration (Pn1) of the primary tumor a decreased M1 and increased M2 polarization in the lymph node sinuses was shown in our study.

According to literature lymph vessel infiltration (L1) and perineural infiltration (Pn1) in oral cancer are associated with the occurrence of lymph node metastasis and indicate an inferior prognosis [45]. Histopathologically defined Pn1 is also associated with an increased risk of local recurrence [46].

In cases with high grading (G3) we also observed a shift towards M2 polarized macrophages in the LN sinuses. Literature shows that high grading is associated with higher incidence of lymph node and distant metastasis [47] and correlates with a poor prognosis of oscc patients [47,48].

Compared to pT1 cases, pT2 cases showed a significantly increased M2 polarization in the interfollicular zone of the lymph nodes. Thus, increased invasiveness is associated with augmented M2 polarization in the regional lymph nodes. According to other published data, the T-status shows a significant correlation with the prognosis of oscc patients [44].

Combining aforementioned outcomes of our study it can be hypothesized that increased malignant behavior of the primary carcinoma influences the immunological situation in the draining lymph nodes. Increased invasiveness and malignancy are associated with immune tolerance-mediating M2 macrophages in the lymph nodes.

However, we did not find any difference regarding the macrophage marker expression in the tumor free lymph nodes of N0 and N + carcinomas. Therefore, it can be hypothesized that there are no general differences concerning the lymph node macrophage polarization in patients free of metastatic tumor growth compared to patients that develop lymph node metastasis. This has to be interpreted considering the results of our previous study analyzing the macrophage polarization in oscc tumor tissue [26]. A correlation of the macrophage polarization of the primary tumor with the occurrence of lymph node metastases was shown [26]. Considering the previous results in conjunction with the results of the current study it can be concluded that the formation of metastases is triggered by the primary tumor and not by the lymph nodes.

Additionally we did not find a difference in macrophage marker expression in tumor free and metastatic lymph nodes. According to this observation, the presence or absence of metastatic tumor growth in lymph nodes seems not to influence local macrophage polarization. This initially astonishing finding is in accordance with other published data [14]. In melanoma patients it could be shown that tumor derived changes in the lymph node immune milieu are independent of the existence of lymph node metastases [14]. T-cell polarization and several immune tolerance markers did not differ between metastatic sentinel lymph nodes and tumor free nodes [49]. However, there are immunological alterations in tumor-associated lymph nodes that prepare them for the acceptance of tumor cells and are a prerequisite for metastatic tumor growth [14,49]. Our data indicate that there might be a similar situation in oral cancer.

The fact that we did not detect a different immune reactivity in tumor free and metastatic lymph nodes also suggests that the tumor causes systemic or at least loco-regionally homogeneous immune tolerance. This finding is analogous to 30 year old studies analyzing the PHA-reactivity in the serum of oscc patients that already indicated a systemic tumor derived immune suppression [8,9].

In addition, the role of the immune system in oscc progression is underlined by the results of a recent study investigating parameters influencing the prognosis of HPV positive oral cancer [28]. The prognosis of HPV positive cases with low levels of tumor infiltrating lymphocytes (TIL) did not differ from HPV negative cases. Whereas, the HPV infected cases with high TIL-levels showed the commonly described superior survival [28]. The HPV infected tumor cells seem to be targets for immune cells and fail to establish a tumor derived immune tolerance. This finding can be understood as a general principle: The prognosis of tumors seems to correlate with the ability of the host defense to target the cancer cells.

It needs to be considered that the markers (CD68, CD11c, CD163 and MRC1) used in this study are not completely macrophage specific. E.g. CD68 is also expressed by fibroblasts and also by some cancer cells [50]. Besides macrophages, CD11c is also expressed by some types of dendritic cells. We tried to overcome this problem as far as possible by counting only cells with a macrophage-like morphology. This was supervised by a pathologist.

The results of the present study also indicate the importance of spatial resolution in the investigation of macrophage polarization in lymph nodes. The benefit of the complete digitalization and virtual microscope systems performed in this study was the ability to exactly mark the different lymph node compartments on all analyzed fields of view and to perform a separate cell counting. Analog studies in other types of carcinoma would be desirable to validate the hypothesis that impaired lymph node macrophage polarization is a general principle of tumor progression or special to oral squamous cell carcinomas.

The association of criteria of increased malignancy and invasiveness of the primary tumor and lymph node M2 polarization might be an expression of a tumor derived peripheral immune tolerance, facilitating further tumor progression.

A therapeutic approach targeting macrophage polarization could be the application of bisphosphonates. Recent studies have shown a beneficial effect of bisphosphonates in early stage breast cancer lacking bone metastasis [51,52]. A possible explanation for this clinical observation could be the recently discovered capability of bisphosphonates to repolarize macrophages from a tumor promoting M2 phenotype to an anti-tumoral M1 phenotype [53]. In a mouse model it could be shown that clinically achievable doses of the bisphosphonate zoledronic acid repolarized tumor associated macrophages towards a full M1 activation state [54]. These data indicate the potential of bisphosphonates to directly target tumor associated macrophages [54].

## Conclusions

Concluding, we can propose the hypothesis that the tumor influences the milieu in the regional lymph nodes and prepares them for the acceptance of metastatic growth by establishing a state of peripheral immune tolerance. However, the decision if the primary tumor establishes metastatic lesions is not made in the lymph nodes, but in the primary tumor.

These considerations should encourage further research analyzing the underlying mechanisms of tumor derived and macrophage mediated peripheral immune tolerance. Breaking immune tolerance – e.g. by using bisphosphonates – would open new perspectives in viral cancer therapy and cancer vaccination strategies, expanding our current treatment options.

## Competing interests

The authors declare that they have no competing interests.

## Authors’ contributions

The authors’ initials are used. FW formulated the hypothesis, interpreted the data and wrote the manuscript. MW applied for grant support (ELAN-Fonds, University of Erlangen), initiated and conducted the study, established and conducted the methods and analytic procedures and contributed significantly to the manuscript. MB helped validating the markers, contributed to the discussion and critically reviewed the manuscript. PH formulated the hypothesis and was involved in the discussion. LD helped establishing the cell counting method and critically reviewed the manuscript. RP, CS, and PM helped with cell counting and critically reviewed the manuscript. JR, KA and FN contributed to the discussion and critically reviewed the manuscript. PM performed the immunohistochemical analysis. All authors read and approved the final manuscript.

## Pre-publication history

The pre-publication history for this paper can be accessed here:

http://www.biomedcentral.com/1471-2407/14/522/prepub

## References

[B1] Shaw RJ Pace-Balzan A Butterworth C Contemporary clinical management of oral squamous cell carcinoma Periodontol 2011 57 1 89 101 10.1111/j.1600-0757.2011.00392.x 21781181

[B2] Scully C Bagan J Oral squamous cell carcinoma: overview of current understanding of aetiopathogenesis and clinical implications Oral Dis 2009 15 6 388 399 19371401 10.1111/j.1601-0825.2009.01563.x

[B3] Warnakulasuriya S Global epidemiology of oral and oropharyngeal cancer Oral Oncol 2009 45 4–5 309 316 18804401 10.1016/j.oraloncology.2008.06.002

[B4] Kumagai K Hamada Y Gotoh A Kobayashi H Kawaguchi K Horie A Yamada H Suzuki S Suzuki R Evidence for the changes of antitumor immune response during lymph node metastasis in head and neck squamous cell carcinoma Oral Surg Oral Med Oral Pathol Oral Radiol Endod 2010 110 3 341 350 20598595 10.1016/j.tripleo.2010.03.030

[B5] Weber W Reuther J Muhling J Ordung R Michel C [Statistical results in patients with squamous cell cancer of the mouth, 1981–1990 patient sample] Fortschr Kiefer Gesichtschir 1992 37 33 36 1639306

[B6] Fan S Tang QL Lin YJ Chen WL Li JS Huang ZQ Yang ZH Wang YY Zhang DM Wang HJ Dias-Ribeiro E Cai Q Wang L A review of clinical and histological parameters associated with contralateral neck metastases in oral squamous cell carcinoma Int J Oral Sci 2011 3 4 180 191 22010576 10.4248/IJOS11068 PMC3469975

[B7] Lingen MW Kalmar JR Karrison T Speight PM Critical evaluation of diagnostic aids for the detection of oral cancer Oral Oncol 2008 44 1 10 22 17825602 10.1016/j.oraloncology.2007.06.011 PMC2424250

[B8] Hyckel P Metzner G Muller P Haroske D Quade R [The significance of immunologic parameters for preoperative prognostication in carcinoma of the mouth] Dtsch Z Mund Kiefer Gesichtschir 1985 9 6 461 468 3869490

[B9] Bier J Nicklisch U [Cellular and humoral immune reactivity in patients with squamous cell carcinoma of the oral cavity] Dtsch Zahnarztl Z 1977 32 10 804 807 269795

[B10] Metelmann HR Hyckel P Podmelle F Oral cancer treatment and immune targets - a role for dendritic cells? J Craniomaxillofac Surg 2012 40 2 103 104 21459014 10.1016/j.jcms.2011.03.009

[B11] Kurahara H Shinchi H Mataki Y Maemura K Noma H Kubo F Sakoda M Ueno S Natsugoe S Takao S Significance of M2-polarized tumor-associated macrophage in pancreatic cancer J Surg Res 2011 167 2 e211 219 19765725 10.1016/j.jss.2009.05.026

[B12] Mantovani A Allavena P Sica A Balkwill F Cancer-related inflammation Nature 2008 454 7203 436 444 18650914 10.1038/nature07205

[B13] Rauser S Langer R Tschernitz S Gais P Jutting U Feith M Hofler H Walch A High number of CD45RO + tumor infiltrating lymphocytes is an independent prognostic factor in non-metastasized (stage I-IIA) esophageal adenocarcinoma BMC Cancer 2010 10 608 21054833 10.1186/1471-2407-10-608 PMC2988756

[B14] Grotz TE Mansfield AS Jakub JW Markovic SN Regional lymphatic immunity in melanoma Melanoma Res 2012 22 1 9 18 22082957 10.1097/CMR.0b013e32834e1f33

[B15] Mao Y Poschke I Wennerberg E de Pico Coana Y Egyhazi Brage S Schultz I Hansson J Masucci G Lundqvist A Kiessling R Melanoma-educated CD14+ cells acquire a myeloid-derived suppressor cell phenotype through COX-2-dependent mechanisms Cancer Res 2013 73 13 3877 3887 23633486 10.1158/0008-5472.CAN-12-4115

[B16] Swartz MA Lund AW Lymphatic and interstitial flow in the tumour microenvironment: linking mechanobiology with immunity Nat Rev Cancer 2012 12 3 210 219 22362216 10.1038/nrc3186

[B17] Watanabe Y Katou F Ohtani H Nakayama T Yoshie O Hashimoto K Tumor-infiltrating lymphocytes, particularly the balance between CD8(+) T cells and CCR4(+) regulatory T cells, affect the survival of patients with oral squamous cell carcinoma Oral Surg Oral Med Oral Pathol Oral Radiol Endod 2010 109 5 744 752 20303300 10.1016/j.tripleo.2009.12.015

[B18] Shah W Yan X Jing L Zhou Y Chen H Wang Y A reversed CD4/CD8 ratio of tumor-infiltrating lymphocytes and a high percentage of CD4 (+) FOXP3 (+) regulatory T cells are significantly associated with clinical outcome in squamous cell carcinoma of the cervix Cell Mol Immunol 2011 8 1 59 66 21200385 10.1038/cmi.2010.56 PMC4002991

[B19] Ino Y Yamazaki-Itoh R Shimada K Iwasaki M Kosuge T Kanai Y Hiraoka N Immune cell infiltration as an indicator of the immune microenvironment of pancreatic cancer Br J Cancer 2013 108 4 914 923 23385730 10.1038/bjc.2013.32 PMC3590668

[B20] Balermpas P Michel Y Wagenblast J Seitz O Weiss C Rodel F Rodel C Fokas E Tumour-infiltrating lymphocytes predict response to definitive chemoradiotherapy in head and neck cancer Br J Cancer 2014 110 2 501 509 24129245 10.1038/bjc.2013.640 PMC3899751

[B21] Costa NL Valadares MC Souza PP Mendonca EF Oliveira JC Silva TA Batista AC Tumor-associated macrophages and the profile of inflammatory cytokines in oral squamous cell carcinoma Oral Oncol 2012 10.1016/j.oraloncology.2012.09.012 23089461

[B22] El-Rouby DH Association of macrophages with angiogenesis in oral verrucous and squamous cell carcinomas J Oral Pathol Med 2010 39 7 559 564 20412402 10.1111/j.1600-0714.2010.00879.x

[B23] Fujii N Shomori K Shiomi T Nakabayashi M Takeda C Ryoke K Ito H Cancer-associated fibroblasts and CD163-positive macrophages in oral squamous cell carcinoma: their clinicopathological and prognostic significance J Oral Pathol Med 2012 41 6 444 451 22296275 10.1111/j.1600-0714.2012.01127.x

[B24] Lu CF Huang CS Tjiu JW Chiang CP Infiltrating macrophage count: a significant predictor for the progression and prognosis of oral squamous cell carcinomas in Taiwan Head Neck 2010 32 1 18 25 19484765 10.1002/hed.21138

[B25] Tong CC Kao J Sikora AG Recognizing and reversing the immunosuppressive tumor microenvironment of head and neck cancer Immunol Res 2012 54 1–3 266 274 22454102 10.1007/s12026-012-8306-6

[B26] Weber M Buttner-Herold M Hyckel P Moebius P Distel L Ries J Amann K Neukam FW Wehrhan F Small oral squamous cell carcinomas with nodal lymphogenic metastasis show increased infiltration of M2 polarized macrophages - An immunohistochemical analysis J Craniomaxillofac Surg 2014 10.1016/j.jcms.2014.01.035 24556525

[B27] Vu HL Sikora AG Fu S Kao J HPV-induced oropharyngeal cancer, immune response and response to therapy Cancer Lett 2010 288 2 149 155 19628331 10.1016/j.canlet.2009.06.026

[B28] Ward MJ Thirdborough SM Mellows T Riley C Harris S Suchak K Webb A Hampton C Patel NN Randall CJ Cox HJ Jogai S Primrose J Piper K Ottensmeier CH King EV Thomas GJ Tumour-infiltrating lymphocytes predict for outcome in HPV-positive oropharyngeal cancer Br J Cancer 2014 110 2 489 500 24169344 10.1038/bjc.2013.639 PMC3899750

[B29] Mantovani A Sica A Locati M New vistas on macrophage differentiation and activation Eur J Immunol 2007 37 1 14 16 17183610 10.1002/eji.200636910

[B30] Mantovani A Biswas SK Galdiero MR Sica A Locati M Macrophage plasticity and polarization in tissue repair and remodelling J Pathol 2013 229 2 176 185 23096265 10.1002/path.4133

[B31] Sica A Mantovani A Macrophage plasticity and polarization: in vivo veritas J Clin Invest 2012 122 3 787 795 22378047 10.1172/JCI59643 PMC3287223

[B32] Hirata Y Tabata M Kurobe H Motoki T Akaike M Nishio C Higashida M Mikasa H Nakaya Y Takanashi S Igarashi T Kitagawa T Sata M Coronary atherosclerosis is associated with macrophage polarization in epicardial adipose tissue J Am Coll Cardiol 2011 58 3 248 255 21737014 10.1016/j.jacc.2011.01.048

[B33] Cao X Shen D Patel MM Tuo J Johnson TM Olsen TW Chan CC Macrophage polarization in the maculae of age-related macular degeneration: A pilot study Pathol Int 2011 61 9 528 535 21884302 10.1111/j.1440-1827.2011.02695.x PMC3292787

[B34] Murray PJ Wynn TA Obstacles and opportunities for understanding macrophage polarization J Leukoc Biol 2011 89 4 557 563 21248152 10.1189/jlb.0710409 PMC3058818

[B35] Cho KY Miyoshi H Kuroda S Yasuda H Kamiyama K Nakagawara J Takigami M Kondo T Atsumi T The Phenotype of Infiltrating Macrophages Influences Arteriosclerotic Plaque Vulnerability in the Carotid Artery J Stroke Cerebrovasc Dis 2012 10.1016/j.jstrokecerebrovasdis.2012.11.020 23273713

[B36] Kawamura K Komohara Y Takaishi K Katabuchi H Takeya M Detection of M2 macrophages and colony-stimulating factor 1 expression in serous and mucinous ovarian epithelial tumors Pathol Int 2009 59 5 300 305 19432671 10.1111/j.1440-1827.2009.02369.x

[B37] Pejnovic N Pantic J Jovanovic I Radosavljevic G Milovanovic M Nikolic I Zdravkovic N Djukic A Arsenijevic N Lukic M Galectin-3 Deficiency Accelerates High-Fat Diet Induced Obesity and Amplifies Inflammation in Adipose Tissue and Pancreatic Islets Diabetes 2013 62 1932 1944 23349493 10.2337/db12-0222 PMC3661611

[B38] Fischer-Posovszky P Wang QA Asterholm IW Rutkowski JM Scherer PE Targeted deletion of adipocytes by apoptosis leads to adipose tissue recruitment of alternatively activated M2 macrophages Endocrinology 2011 152 8 3074 3081 21693678 10.1210/en.2011-1031 PMC3138241

[B39] Aron-Wisnewsky J Tordjman J Poitou C Darakhshan F Hugol D Basdevant A Aissat A Guerre-Millo M Clement K Human adipose tissue macrophages: m1 and m2 cell surface markers in subcutaneous and omental depots and after weight loss J Clin Endocrinol Metab 2009 94 11 4619 4623 19837929 10.1210/jc.2009-0925

[B40] Hasan D Chalouhi N Jabbour P Hashimoto T Macrophage imbalance (M1 vs. M2) and upregulation of mast cells in wall of ruptured human cerebral aneurysms: preliminary results J Neuroinflammation 2012 9 222 22999528 10.1186/1742-2094-9-222 PMC3488554

[B41] van Putten SM Ploeger DT Popa ER Bank RA Macrophage phenotypes in the collagen-induced foreign body reaction in rats Acta Biomater 2013 6502 10 10.1016/j.actbio.2013.01.022 23376130

[B42] Lan C Huang X Lin S Huang H Cai Q Wan T Lu J Liu J Expression of M2-Polarized Macrophages is Associated with Poor Prognosis for Advanced Epithelial Ovarian Cancer Technol Cancer Res Treat 2012 10.7785/tcrt.2012.500312 23289476

[B43] Herwig MC Bergstrom C Wells JR Holler T Grossniklaus HE M2/M1 ratio of tumor associated macrophages and PPAR-gamma expression in uveal melanomas with class 1 and class 2 molecular profiles Exp Eye Res 2013 107 52 58 23206928 10.1016/j.exer.2012.11.012 PMC3556238

[B44] O'Brien CJ Lauer CS Fredricks S Clifford AR McNeil EB Bagia JS Koulmandas C Tumor thickness influences prognosis of T1 and T2 oral cavity cancer–but what thickness? Head Neck 2003 25 11 937 945 14603454 10.1002/hed.10324

[B45] Lanzer M Gander T Kruse A Luebbers HT Reinisch S Influence of histopathologic factors on pattern of metastasis in squamous cell carcinoma of the head and neck Laryngoscope 2013 10.1002/lary.24458 24254388

[B46] Fagan JJ Collins B Barnes L D'Amico F Myers EN Johnson JT Perineural invasion in squamous cell carcinoma of the head and neck Arch Otolaryngol Head Neck Surg 1998 124 6 637 640 9639472 10.1001/archotol.124.6.637

[B47] Fortin A Couture C Doucet R Albert M Allard J Tetu B Does histologic grade have a role in the management of head and neck cancers? J Clin Oncol 2001 19 21 4107 4116 11689578 10.1200/JCO.2001.19.21.4107

[B48] Carinci F Pelucchi S Farina A De Franciscis G Calearo C Extension as a prognostic factor in oropharyngeal cancer: largest mucosal dimension compared with number of (sub) sites involved Br J Oral Maxillofac Surg 1998 36 6 440 445 9881786 10.1016/s0266-4356(98)90460-0

[B49] Mansfield AS Holtan SG Grotz TE Allred JB Jakub JW Erickson LA Markovic SN Regional immunity in melanoma: immunosuppressive changes precede nodal metastasis Mod Pathol 2011 24 4 487 494 21151098 10.1038/modpathol.2010.227

[B50] Beranek JT CD68 is not a macrophage-specific antigen Ann Rheum Dis 2005 64 2 342 343 author reply 343–344 15647451 PMC1755344

[B51] Coleman R Gnant M Morgan G Clezardin P Effects of bone-targeted agents on cancer progression and mortality J Natl Cancer Inst 2012 104 14 1059 1067 22752060 10.1093/jnci/djs263

[B52] Ben-Aharon I Vidal L Rizel S Yerushalmi R Shpilberg O Sulkes A Stemmer SM Bisphosphonates in the adjuvant setting of breast cancer therapy–effect on survival: a systematic review and meta-analysis PLoS One 2013 8 8 e70044 23990894 10.1371/journal.pone.0070044 PMC3753308

[B53] Rogers TL Holen I Tumour macrophages as potential targets of bisphosphonates J Transl Med 2011 9 177 22005011 10.1186/1479-5876-9-177 PMC3215187

[B54] Coscia M Quaglino E Iezzi M Curcio C Pantaleoni F Riganti C Holen I Monkkonen H Boccadoro M Forni G Musiani P Bosia A Cavallo F Massaia M Zoledronic acid repolarizes tumour-associated macrophages and inhibits mammary carcinogenesis by targeting the mevalonate pathway J Cell Mol Med 2010 14 12 2803 2815 19818098 10.1111/j.1582-4934.2009.00926.x PMC3822730

